# Right perirenal urinoma and urinothorax in an infant after neonatal ablation of posterior urethral valve: A rare complication diagnosed by pleural aspiration and treated with perinephric drainage: a case report

**DOI:** 10.1186/s13256-024-04634-9

**Published:** 2024-06-29

**Authors:** AbdulRahman AlDaithan, Mohamed Basuni, Mohamed ElSeadawy

**Affiliations:** Pediatric Intensive Care Unit, Department of Pediatrics, Farwaniya Hospital, Sabah Al Nasser Area, Kuwait

**Keywords:** Urinothorax, Urinoma, PUV, Pleural effusion, Case report

## Abstract

**Objective:**

Urinothorax and urinoma are rare complications of obstructive uropathy. They might occur due to persistent high back pressure on the renal parenchyma. Urinothorax usually arises while the obstruction exists; in contrast to our case, the child presented after being operated on. He had falsely high creatinine before the operation, which was later explained by creatinine recirculation.

**Clinical presentation and intervention:**

We are reporting an uncommon case of late presentation of ruptured urinoma in a 2-month-old Kuwaiti male. It led to urinothorax/uroperitoneum that caused respiratory distress and was associated with creatinine recirculation, requiring retroperitoneal perinephric catheter insertion. The child had recovered and was discharged home.

**Conclusion:**

A high index of suspicion is required to diagnose urinothorax, especially in patients with a history of obstructive uropathy. Aspiration of the pleural effusion will guide you to reach the diagnosis. Creatinine recirculation is rarely described in the literature. Having a patient with urinothorax/uroperitoneum should raise the suspicion of falsely elevated creatinine levels.

## Introduction

Urinothorax is defined as an accumulation of urine in the pleural space, a rare etiology for pleural effusions [1]. It can occur due to variable reasons, including trauma, obstructive uropathy, lithotripsy complication, malignancy, gravid uterus, polycystic kidney disease, or post-renal transplant [2].

To establish the diagnosis, you need a pleural-to-urine creatinine ratio that is more than 1 [3]. On correcting the causative culprit, urinothorax will be corrected. We report a case of urinothorax that occurred after posterior urethral valve surgery.

## Case report

A 2-month-old Kuwaiti preterm boy underwent posterior urethral valve (PUV) ablation at the age of 5 days then stayed in the neonatal intensive care unit (NICU) for weight gain and observation after the surgery till full recovery. Two weeks later after discharge from NICU, he presented with two days history of respiratory distress, right-sided flank swelling, lethargy, vomiting, and mottled skin. He was shifted to the Pediatric Intensive Care Unit (PICU) for respiratory support.

**Fig. 1:**
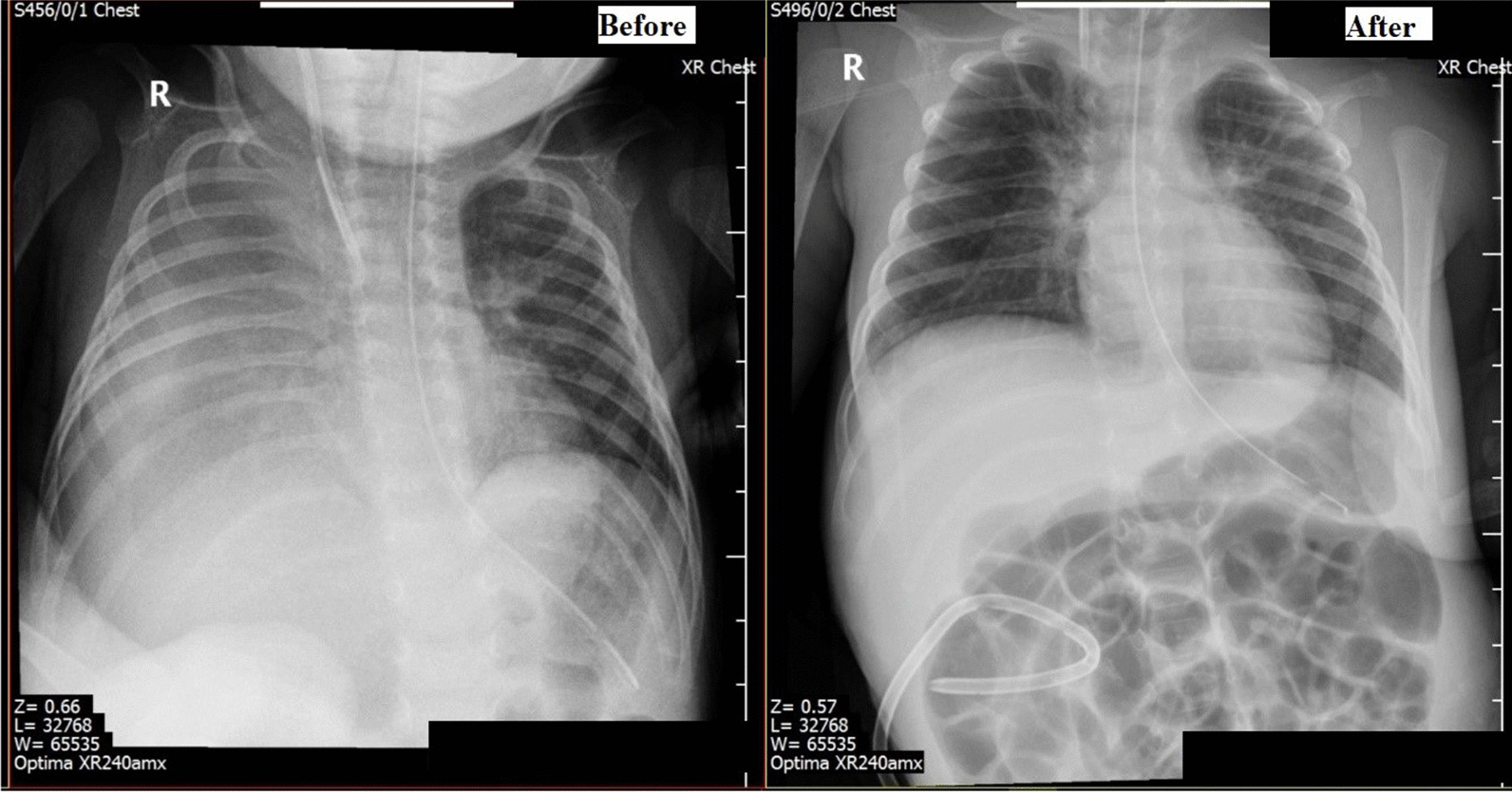
Chest X-ray showing before and after perinephric insertion with right-sided pleural effusion improvement

**Fig. 2:**
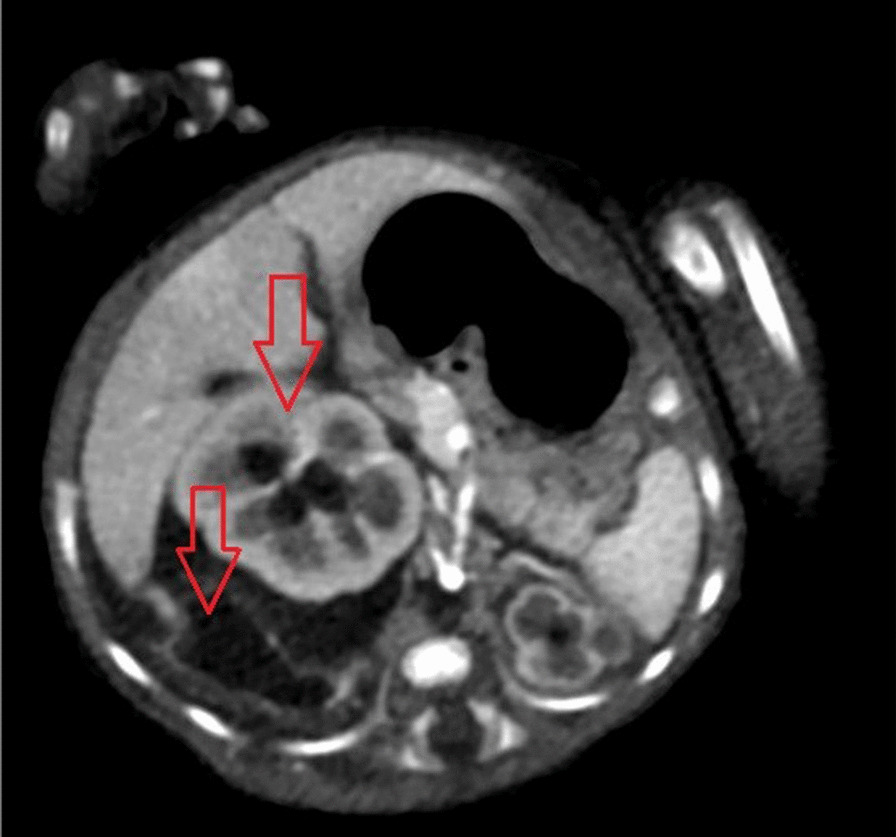
Abdomen CT shows right-side hydronephrosis with fluid leakage retroperitoneum (Arrows)

His blood work results confirmed the diagnosis of acute renal failure (Table 1). His chest x-ray showed right-side pleural effusion (Fig. 1). A septic work-up was performed, including a pleural tab to rule out infectious causes, results came as follows: the CBC [WBCs 32.2*10^9^/L, neutrophils 61%, lymphocytes 26.3%], CRP 70mg/L, PCT 0.06ng/ml, blood, CSF, ETT, pleural fluid and urine cultures revealed no growth. Antibiotics were started empirically but were discontinued after all cultures were negative. Ultrasound of the abdomen and pelvis revealed mild free pelviabdominal fluid plus hydroureteronephrosis with a picture suspecting of urinoma associated with bilateral pleural effusion more on the right side. A CT scan of the abdomen and pelvis with contrast revealed leakage in the right perirenal, perihepatic, and subhepatic regions (Fig. 2).

**Table 1:** Patient’s electrolyte and renal function at different stages

Kidney functions and electrolytes–blood sample (Unit)	On admission	24 h pre-urinoma drain	24 h post-urinoma drain	on discharge from PICU
Urea (mmol/L)	11.4	5.6	2.4	3.5
Creatinine (umol/L)	341	191	32	25
Sodium (mmol/L)	128	142	142	137
Potassium (mmol/L)	7.4	5.9	5.9	4.8
Calcium (mmol/L)	2.2	2.7	2.7	2.2
Phosphate (mmol/L)	2.5	2	2	1.19
Bicarbonate (mmol/L)	17	18	22	20

The right-side Pleural fluid tapping results revealed glucose 4.4 mmol/L and creatinine 140 umol/l, while serum creatinine was 132 μmol/l.

The pediatric urologist team inserted a retroperitoneal pigtail catheter to drain the fluids surrounding the urinoma, and it was kept till the ruptured urinoma regressed and healed by itself. Initially, they tried to insert a percutaneous nephrostomy tube but failed due to the small size of the kidney. Serum creatinine level dramatically improved after the procedure.

He was initially kept on high-flow nasal cannula (HFNC) due to respiratory distress secondary to the urinothorax. After inserting the retroperitoneal drainage catheter surrounding the urinoma, draining 234 ml of urine, and diverting the urine from accumulating inside the peritoneum and the pleura, pleural effusion regressed. His respiratory effort improved dramatically with the regression of the uroperitoneum and urinothorax. He was successfully removed from the HFNC.

The patient was discharged home with a two weeks follow-up in the pediatric urology outpatient department to assess the ruptured site and to remove the retroperitoneal drainage catheter. Subsequent to the initial follow up visit, he underwent multiple follow-up evaluations.

During the latest visit, occurring at the age of 11 months, a kidney ultrasound revelaed grade 2 hydronephrosis in the right kidney, alongside poor corticomedullary differentiation in the left kidney. Notably, the patient did not necessitate hospitalization and there were no recurrence of the perirenal urinoma or the urinothorax (Table 2).

**Table 2:** Patient time line and follow up ultrasound findings

Age	Events	Findings
5 days	PUV operated	
39 days	Discharged from NICU	
60 days	Admitted to PICU	
67 days	Retroperitoneal drainage catheter inserted	
70 days	Discharged from PICU	
97 days	Pigtail removal	
3 months	MCUG	No evidence of vesicoureteric reflux on both sides with bladder capacity 30 cc and mildly dilated posterior aspect of urethra
5 months	Follow up utrasound	Right Kidney: 76 mm, kidney parenchyma and collecting system appear normal, however mild caliectasis is noted Left Kidney: 33mm, kidney parenchyma appears normal with mild pelvic fullness Bladder: Wall thickness: normal Ureters: Not dilated Impression: Small‐sized left kidney with mild pelvic fullness. Right mild caliectasis
8 months	Follow up utrasound	RT kidney: 66 mm with Grad I hydronephrosis Pelvic width 8 mm pelvic volume 0.6 ml Parenchyma thickness 11 mm Left kidney: 29 mm thin poorly differentiated renal parenchyma with small lower pole 6 × 4.4 mm cyst Urinary bladder: Inadequately filled with 10 ml clear urine Ureters: RT lower end ureter 2.7 mm Left lower end ureter 3.9 mm Impression: RT hydronephrosis. Small left kidney Prominent both lower end ureters
11 months	Follow up utrasound	Right Kidney: 69 mm, Grade II Hydronephrosis. Pelvis width: 8mm, volume: 0.65 ml Left Kidney: 27 mm, poor corticomedullary differentiation (CMD) with prominent pelvis Bladder: Normal wall not thick Ureters: Not dilated Impression: Right hydronephrosis. Small‐sized left kidney with poor CMD and prominent pelvis

## Discussion

Urinothorax was first described in the 1950s by France and Back, followed by Corriere et al. in 1968 when they noticed two cases of unilateral pleural effusion associated with hydronephrosis [4].

It could result from complications of urinary tract obstruction, retroperitoneal inflammation, trauma, or surgical procedures [5, 6]. Most of the cases of urinothorax manifest with an ipsilateral urinoma. However, there are cases of bilateral and contralateral urinothorax [7].

Due to the rarity of the disease, the true prevalence of urinothorax in pediatric patients with obstructive uropathy is unknown.

Urinothorax can also occur indirectly through the lymphatic system; urine will migrate from the intra-abdominal into the intra-thoracic space through the lymphatic system; then, due to pressure gradient, the fluid can leak back into the pleural space or even the venous system which might be the explanation for the recirculating creatinine in our case [4].

The most common etiologies of the Urinothorax are obstructive uropathy [7]. There are many causes of urinary tract obstruction. A posterior urethral valve is the most common cause of outflow urinary tract obstruction in male infants [8]. Patients with posterior urethral valve concomitantly might have unilateral or bilateral vesicoureteral reflux with dysplastic nonfunctioning kidneys. The dysplastic nonfunctioning kidneys will have limited uptake of the contrast making interpretation of radiographic studies questionable, especially when looking for urinoma and urinothorax [7, 9].

The diagnosis of urinothorax is made via testing the plural fluids [4]. The most essential biomarker to make the diagnosis is the fluid creatinine to serum creatinine ratio, which is required to be higher than 1 [10–12]. Thoracentesis may relieve the symptoms of urinothorax temporarily until treating the underlying cause [4].

Serum Creatinine levels might not be accurate in patients with urinothorax or uroperitoneum since it is theorized that the urine is being absorbed by lymphatics, which might eventually affect the creatinine levels in the venous system, especially if the rate of reabsorption is higher than the excretion of creatinine [8]. As for prognosis and outcome, treating the underlying uropathy alone had a favorable outcome even without a thoracic drainage [4].

## Conclusion

Urinothorax and uroperitoneum are rare complications of obstructive uropathy. They might lead to recirculation and persistently high levels of creatinine. A high index of suspicion is required to diagnose urinothorax. Management should involve releasing the obstruction and draining the fluids until the affected site heals.

## Data Availability

The data supporting the findings of this study are available within the article.

## Abbreviations

PUV: Posterior urethral valve
PICU: Pediatric Intensive Care Unit
HFNC: High flow nasal cannula
